# In vitro anti-tuberculosis effect of probiotic *Lacticaseibacillus rhamnosus* PMC203 isolated from vaginal microbiota

**DOI:** 10.1038/s41598-022-12413-z

**Published:** 2022-05-18

**Authors:** Md Abdur Rahim, Hoonhee Seo, Sukyung Kim, Hanieh Tajdozian, Indrajeet Barman, Youngkyoung Lee, Saebim Lee, Ho-Yeon Song

**Affiliations:** 1 Probiotics Microbiome Convergence Center, Soonchunhyang University, Asan, 31538 Chungnam Korea; 2 Department of Microbiology and Immunology, School of Medicine, Soonchunhyang University, Cheonan, 31151 Chungnam Korea

**Keywords:** Microbiology, Antimicrobials

## Abstract

*Mycobacterium tuberculosis* (*M. tb*), the etiological agent of tuberculosis (TB), poses a severe challenge for public health and remains the number one cause of death as a single infectious agent. There are 10 million active cases of TB per year with 1.5 million deaths, and 2–3 billion people are estimated to harbor latent *M. tb* infection. Moreover, the emergence of multi-drug-resistant (MDR), extremely-drug-resistant (XDR), and the recent totally drug-resistant (TDR) *M. tb* is becoming a global issue that has fueled the need to find new drugs different from existing regimens. In these circumstances, probiotics can be a potential choice, so we focused on developing them as an anti-tuberculosis drug candidate. Here, we report the anti-tubercular activities of *Lacticaseibacillus rhamnosus* PMC203 isolated from the vaginal microbiota of healthy women. PMC203 exhibited a promising intracellular killing effect against both drug-sensitive and resistant *M. tb* infected murine macrophage cell line RAW 264.7 without showing any cytotoxicity. Additionally, it also inhibited the growth of *M. tb* under broth culture medium. PMC203 did not cause weight change or specific clinical symptoms in a 2-week repeated oral administration toxicity test in a guinea pig model. Here, we also found that PMC203 induces autophagy in a dose dependent manner by increasing the signal of well-known autophagy gene markers, suggesting a possible intracellular killing mechanism.

## Introduction

Tuberculosis (TB), caused by the etiological agent of *Mycobacterium tuberculosis* that remains the world’s oldest known deadly infectious disease in humans, considering as one of the top ten leading causes of death globally^1^. According to the World Health Organization (WHO) report 2021, 10 million people were infected with tuberculosis bacteria in 2020, with 1.3 million TB deaths among Human immunodeficiency Virus (HIV)-negative people, and an additional 214,000 deaths among people who have HIV^2^. The report also shows that 71% of people diagnosed with pulmonary TB were tested to be drug resistant, up from 61% in 2019 and 50% in 2018^2^. The co-occurrence of TB with HIV and an inadvertent resistance mechanism developed by *M. tuberculosis* against existing antagonist which facilitate the emergence of multi-drug resistant (MDR) TB, extensive-drug-resistant (XDR) TB, and totally-drug-resistant (TDR) TB, raise serious concerns and investigate a requirement for urgent attention to repress this deadly disease^3^. Nowadays, the emergence of drug-resistant TB has become a public health threat worldwide^4^. Moreover, current TB treatment involves 6–9 months of course with first line drugs, and it can be even 18–24 months for MDR-TB treatment in combination with second line drugs which are less effective, more toxic, and more expensive than the first line drugs^5–7^. Therefore, it is urgently needed to emphasize the development of new anti-tuberculosis drug to combat this deadly menace.

The WHO identifies probiotics as “Living microorganisms that when administered in adequate amounts as a part of food, confer a health benefit on the host” and include single or multiple strains of live or dead, in combination with fermentable foods, pills, powders, and liquid drops^8^. Probiotics rebalance intestinal microbiota distribution, management of metabolic syndromes, prevention of gastrointestinal diseases, and promote systemic immune responses by activating distinct immune modulatory mechanisms^9^. Several studies reported that probiotics have had beneficial impact on irritable bowel syndrome, wound healing, allergens, diabetes, cancer eczema, atopy, inflammation, psychological stress, hepatic encephalopathy, gastrointestinal disorder, and pathological neonatal jaundice^10,11^.

Moreover, probiotics are used as an immunomodulator that reduces the incidence and severity of infectious diseases. Several studies reported the effect of probiotics against infectious diseases caused by bacteria like *Helicobacter pylori*, *Salmonella*, enterotoxigenic *E. coli*^8^, *Vulvovaginal candidiasis*^12^, *Streptococcus pneumoniae*^13^, and *Campylobacter* sp.^14^.

In recent years, the bactericidal activity of probiotics has extended to suppress numerous antibiotic-resistant superbugs^15^, including Carbapenem-resistant *Enterobacteriaceae* (CRE)^16^, Multidrug-resistant *Acinetobacter baumannii*^17^, Multidrug-resistant *Pseudomonas aeruginosa*^18^, Vancomycin-resistant *Enterococcus* (VRE)^19^, and Methicillin-resistant *Staphylococcus aureus* (MRSA)^20^. Probiotics are even being applied to viral infections such as HIV and SARS-CoV-2^21,22^.

Regarding this, the effect of probiotics on tuberculosis has not been enormous. Therefore, the primary objective of the present study is to investigate the effects of probiotics on the reduction of *M. tb* load in the macrophage Raw cell 264.7 cell lines. This study found that the probiotic strain *Lacticaseibacillus rhamnosus* PMC203 possesses an anti-tuberculosis effect and induces autophagy.

## Methods

### Isolation of test candidate probiotic strains

Test candidate probiotics were isolated from vaginal fluid and different traditional fermented foods. Female vaginal fluid samples were collected with written consent from 33 healthy premenopausal women (18–50 years). This study was approved by the Soonchunhyang University Cheonan Hospital (SCH) Ethics Committee (eIRB No. 2019-10-017-005) and all methods were performed in accordance with the declaration of Helsinki (1964)^23^. Other probiotic strains were isolated from Korean traditional fermented foods (kimchi and vinegar) in the laboratory of Soonchunhyang University. Upon receiving the samples in the lab, we diluted them, streaked them onto MRS agar plates, and incubated them in an incubator at 37 °C for 2 days. The following day, single colonies were cultured in MRS broth and incubated overnight in a shaking incubator. Subsequently, optical density (OD) of the bacterial culture was measured using a spectrophotometer (DR 1900, Hatch, USA) at OD_600nm_, and sent them to the 16S rRNA sequencing company (Biofact, Korea) for identification.

### Identification of probiotic strains by 16S rRNA gene sequencing

The probiotics strain was identified through 16S rRNA gene sequencing technology. Following the extraction of DNA, amplification was performed by PCR using the pair of primers 27F (5′-AGA GTT TGA TCC TGG CTC AG-3′) and 91 1492R (5′-GGT TAC CTT GTT ACG ACT T-3′). Next, an ABI PRISM 3730XL DNA analyzer (Applied Biosystems, USA) was used for the purification and sequencing of the amplified PCR product, and the resulting sequencing data were then compared to the National Center for Biotechnology Information (NCBI) GenBank database using BLAST (basic local alignment search tool).

### Preparation of cell extract of test candidate probiotic strains

All candidate probiotic strains were cultivated in MRS broth (BD Difco, USA) and incubated overnight at 37 °C (BioFree, Korea). The following day, OD of the bacterial culture growth was measured, then centrifugation (Combi R515, Hanil Scientific, Inc. Korea) was performed at 4000 rpm for 10 min, followed by washing them three times with 0.85% NaCl solution. After that, 1 ml of 0.85% NaCl solution was added for the resuspension of the pellet and transferred to the Lysing Matrix B tube (MP Biomedicals, USA) for the mechanical lysis of the bacterial cells using a homogenizer (FastPrep-24 5G, MP Biomedicals, USA), followed by supernatant was collected as cell extract for the anti-mycobacterial test.

### Cell culture and bacteria preparation

Murine macrophage cell line RAW 264.7 (KCLB 40071) was purchased from the Korean Cell Line Bank (KCLB, Korea) and maintained in Dulbecco’s Modified Eagle Medium (DMEM, Gibco, USA) supplemented with 10% fetal bovine serum (FBS) (Gibco, USA) and 1% antibiotics (100 U/ml of penicillin and 100 µg/ml of streptomycin) (HyClone, USA) incubated at 37 °C with a humidified 5% CO_2_ atmosphere. *M. tb* H37Rv (ATCC 27294) was purchased from the American Type Culture Collection (ATCC, USA), and XDR *M. tb* (KMRC 00203-00197) was purchased from the Korean Mycobacterium Resource Center (KMRC, Korea). Middlebrook 7H9 broth (BD Difco, USA) supplemented with 10% OADC (oleic acid-albumin-dextrose-catalase) (BD Difco, USA) and 0.5% Tween 80 (Sigma-Aldrich, USA) was used for the cultivation of *M. tb*. All experiments using *M. tb* were conducted in an Animal Biosafety Level 3 Laboratory of Soonchunhyang University (ABSL-3, KDCA-20-3-04).

### Intracellular anti-mycobacterial activity

#### Intracellular anti-mycobacterial activity test by acid-fast bacilli staining

Monolayers of RAW 264.7 cells (2 × 10^5^ cells/ml) were cultured in 2-well cell culture slides (SPL Life Sciences, Korea), incubated at 37 °C with a humidified 5% CO_2_ atmosphere. After reaching the confluency of approximately 75–85%, cells were washed with phosphate buffer saline (1 × PBS) and exposed to *M. tb* (H37Rv or XDR) at a multiplicity of infection (MOI) of 10 for 2 h. Next, the cells were washed carefully, and probiotics extract in drug-free medium was added and incubated again. 3 days later, the cells were washed with 1 × PBS for three times and acid-fast bacilli (AFB) staining was performed to observe with the light microscope (AX10, Carl Zeiss, Germany).

#### Intracellular anti-mycobacterial activity test by CFU assay

For assessing the anti-mycobacterial activities by the CFU method, macrophage cell monolayers were seeded in a 96-well plate in which the volume of each well was 200 µl. This test was similar to the test described in the earlier section, where other conditions such as cell type, cell density, infection conditions, and incubation time were the same. After 3 days of incubation, the cells were washed with 1 × PBS and the cell lysates were prepared by using autoclave distilled water, and then the viable bacilli released from the cells was enumerated by plating serial dilutions (tenfold) of the cell lysate on 7H10 agar medium (BD Difco, USA) plates. Viable bacilli were then calculated 2–3 weeks later by CFU assay.

### Anti-mycobacterial susceptibility assay

#### Resazurin assay

The anti-mycobacterial activity of the probiotic strain was evaluated using resazurin assay. *M. tb* strains of H37Rv and XDR inocula (1 × 10^5^ CFU/ml) were prepared in a 96-well plate, treated with predetermined concentrations of probiotic extract, and incubated at 37 °C. After 7 days of incubation, 20 µl of freshly prepared 0.2% resazurin solution was added to each well and incubated additionally up to 48 h. Following the additional incubation, fluorescence readings were taken at 570 and 600 nm using a Victor Nivo Multiplate reader (Perkin Elmer, USA).

#### Luminescent microbial cell viability assay

The number of viable bacilli cells was quantified in a probiotic extract culture with luminescent cell viability assay to measure ATP reduction, following the protocol^24^. The luminescent microbial cell viability assay was similar to the resazurin assay. In this test, BacTiter-Glo reagent was used to assay microbial cell viability. Other conditions were similar to the resazurin assay. After 7 days of incubation, 50 µl *M. tb* cells were collected from each well and thoroughly resuspended in 50 µl freshly prepared BacTiter-Glo reagent and incubated additionally at room temperature for 10 min on an orbital shaker. After incubation, the luminescence readings were taken using a Victor Nivo Multi-plate reader (Perkin Elmer, USA).

#### CFU enumeration assay

An enumeration test of the CFU was also used to test the direct killing effect of the probiotic extract against *M. tb*. This test is similar to the resazurin assay and luminescent microbial cell viability assay, as described earlier. After 7 days of incubation, cell suspensions were diluted and spread onto 7H10 agar plates. Viable bacilli were then calculated after 2–3 weeks.

### Anti-mycobacterial activity in co-culture conditions

The growth inhibition activities of probiotics against *M. tb* were investigated by the co-culture method. The probiotics strain (1 × 10^6^ CFU/ml) and *M. tb* strain (1 × 10^8^ CFU/ml) were mixed in which the broth medium used consisted of 10% MRS broth and 90% 7H9 broth. We adjusted the pH of *M. tb* culture broth at 5.1 using hydrochloric acid (Sigma-Aldrich). Initially, the pH of *M. tb* culture broth was 6.6. In the same way, the pH of co-culture broth was also adjusted at 6.7 using sodium hydroxide (Sigma-Aldrich). The initial pH of co-culture broth was 5.2. We adjusted the pH of *M. tb* culture broth and co-culture broth separately to verify whether the acidic condition was created by the probiotic culture itself. The entire culture broths were then incubated at 37 °C for 2 weeks in a shaker incubator. During the incubation period, the CFU of *M. tb* and pH of the culture broths were checked on days 0, 4, 8, and 12.

### Cell viability assay

The cytotoxicity screening of probiotics extract against RAW 264.7 cells was evaluated using trypan blue assay. Briefly, monolayers of RAW 264.7 cells (2 × 10^5^ cells/ml) were cultured onto 2-well culture slides (SPL life sciences) overnight. After reaching approximately 75–85% confluency, the cells were exposed to probiotics extract in new culture media for 24 h. Subsequently, cells were washed, stained with trypan blue (Gibco), and counted under an optical microscope (AX10, Carl Zeiss, Germany) using a hemocytometer (Marienfeld). The viability of the cells was also determined using EZ-Cytox cell viability assay kit solution (DoGenBio, Korea) in which cells were cultured in a 96-well plate, treated with probiotic extract, and incubated. Following incubation, 20 µl of WST solution was added, incubated for 2 h and cytotoxicity was evaluated by measuring the absorbance at 570 nm using a Victor Nivo Multiplate reader (Perkin Elmer, USA). Furthermore, cell viability and morphology was also observed using methylene blue stain (Dagatron, Korea).

### Autophagy detection assay

According to the manufacturer’s protocol, autophagy induction was evaluated using a commercial autophagy detection kit (Abcam, USA). Macrophage 264.7 RAW cells (2 × 10^5^ cells/ml) were seeded in a 96-well plate and incubated overnight. The next day, confluency (approximately 75–85%) was checked, washed, treated with probiotic extract at different predetermined concentrations along with positive and negative control, and incubated for 24 h. In the meantime, the cells were also exposed to H37Rv and treated with PMC203. After that, the cells were washed with 1 × Assay Buffer, treated with a mixture of green detection reagent and nuclear stain mixture, and further incubated at 37 °C for 30 min. Subsequently, the cells were carefully washed using 1 × Assay Buffer, and the Green Detection signal was measured at 480 nm and 530 nm using a Victor Nivo Multi-plate reader (Perkin Elmer, USA).

### Real-time PCR for autophagy gene expression analysis

Total RNA was isolated from probiotic treated RAW 264.7 cells with a kit of RNA protection bacteria reagent (Qiagen, Germany) described by the manufacturer’s protocol to observe the autophagy gene expression. Next, the total RNA integrity was checked by agarose gel electrophoresis and quantified by a Qubit Fluorometer (Invitrogen, USA) using a Qubit RNA Assay kit (Thermo fisher, USA). RNA samples were then reverse transcribed to cDNA with a kit of cDNA synthesis (Bio-Rad, USA), and Real-time PCR was carried out using the SYBR Green Supermix Kit (Bio-rad, USA) with a CFX96 Real-Time PCR detection system following the instructions provided by the company (Applied Biosystems, USA). The values of target gene expression were standardized for the endogenous control gene, glyceraldehyde 3-phosphate dehydrogenase (GAPDH), using the comparative Ct method described previously^25^. The primer pair sequences^9^ are listed in the Supplementary Table S4.

### Acute oral dose toxicity test of probiotic strain in guinea pig model

The guinea pigs used in this study were adult males weighing from 1000 to 1200 g. The total number of animals were 10 and they were randomly divided into a treatment group and a control group where each group consisted of 5 animals. They were housed in an environment-controlled barrier room. The animals were monitored on a 12-h cycle of light and darkness, with temperatures ranging from 20 to 25 °C and relative humidity between 30 to 70 per cent. All animals had free access to standard commercial food and drinkable water ad libitum*.* The candidate probiotic strain PMC203 was cultured, washed, and resuspended with 0.85% NaCl solution to adjust the bacterial number to 2 × 10^6^ CFU/kg of body weight. The treatment group was orally administered the probiotic solution once daily for 5 days per week for 2 weeks, while the control group received only 0.85% NaCl solution under the same conditions. The animals were observed for clinical signs, mortality, and body weight changes within the dosing period. This animal experiment was conducted at Soonchunhyang University PMC Lab, registered as an animal testing facility (KFDA 657) as per the guidelines for Drug Safety Testing provided by the Ministry of Food and Drug Safety (Notice No. 2015-82) and following the enforcement regulations of the Act on Laboratory Animals licensed as ABSL-2 (LML 20-591). The animal experiment protocol for this study was examined and approved by the Soonchunhyang Institutional Animal Care and Use Committee (IACUC) (Approval number SCH21-0033) and the experimental data is reported in accordance with ARRIVE guidelines.

### Whole-genome sequencing of probiotics strain

Whole-genome sequencing (WGS) analysis was conducted to determine the strain level of our candidate probiotics strain. Genomic DNA (gDNA) was extracted with a mini-kit of QIAamp DNA (Qiagen, Germany) in accordance with the manufacturer’s instructions and was sent to a commercial WGS service company (Chunlab Inc. Seoul, Korea). PacBio sequencing data was compiled with PacBio SMRT Analysis 2.3.0 using the HGAP2 protocol, and contigs resulting from PacBio sequencing were subsequently circulated using Circlator 1.4.0 (Sanger Institute, UK). Protein coding sequences (CDSs) were predicted with Prodigal 2.6.2^26^ and grouped according to roles regarding orthologous groups (EggNOG; http://eggnogdb.embl.de). tRNAscan-SE 1.3.1 was used to search genes encoding tRNAs^27^. rRNAs and other noncoding RNAs were searched by covariance model searches using the Rfam 12.0 database^28^. The OrthoANIu algorithm-based Average Nucleotide Identity (ANI) calculator (https://www.ezbiocloud.net/tools/ani) was used for the comparison of prokaryotic genome sequences^29^.

## Results

### In-vitro screening of test candidate probiotics with anti-mycobacterial effect

**Figure 1:**
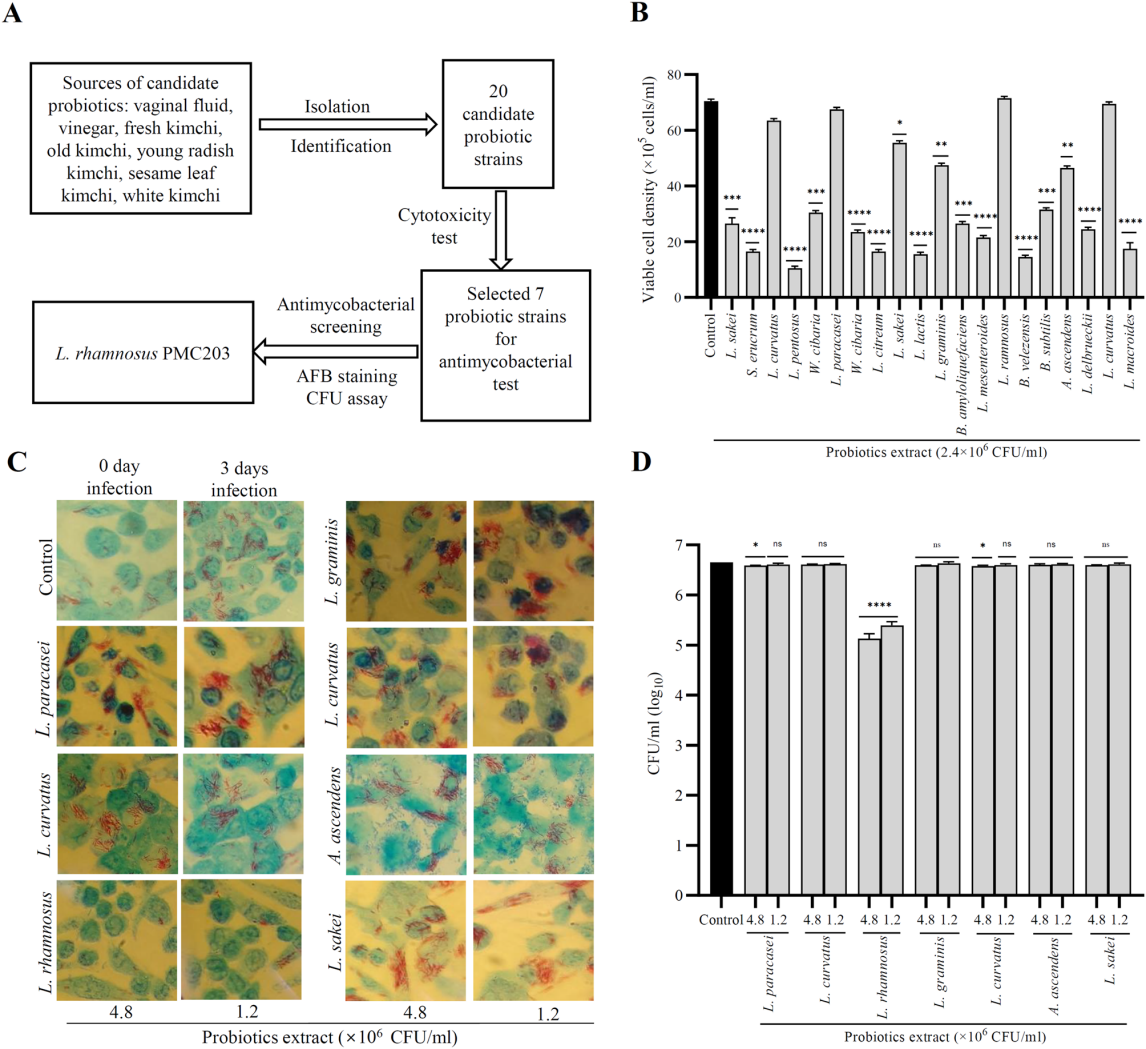
Anti-mycobacterial screening of candidate test probiotics. (**A**) Schematic diagram of the study. (**B**) Monolayers of macrophage cells were treated with the extract of test candidate probiotics and viability was checked using the trypan blue assay. Based on the viability, 7 probiotic strains were selected for anti-mycobacterial screening by (**C**) acid-fast bacilli staining and (**D**) CFU assay. These experiments were carried out in triplicate and statistical significance with controls was analyzed using Graph Pad Prism 9.1.1, one-way ANOVA (*p < 0.05, **p < 0.01, ***p < 0.001, ****p < 0.0001). Control images were taken at days 0 and 3 of infection, while treated cells images were taken after 3 days of incubation (left, 4.8 × 10^6^ CFU/ml; right, 1.2 × 10^6^ CFU/ml).

Figure 1 showed the in vitro anti-mycobacterial screening procedures of the test candidate probiotics. Overall study diagram is depicted in Fig. 1A from isolation of test candidate probiotics to anti-mycobacterial screening. Cytotoxicity of 20 test candidate probiotics isolated from different sources was evaluated using trypan blue assay (Fig. 1B). The name of the isolated test candidate probiotic strains and their sources are enlisted in Supplementary Table S1. The freshly prepared test probiotics’ extract was used to treat the 264.7 RAW cells at the concentration of 2.4 × 10^6^ CFU/ml. Among the 20 candidate test probiotics, 7 strains (*L. paracasei*, *L. curvatus*, *L. rhamnosus*, *L. graminis*, *L curvatus*, *A. ascendens*, and *L. sakei*) did not show significant toxicity to cells and viability was more than 65%. Based on the viability, these 7 probiotic strains were then selected for anti-mycobacterial screening test. To do that, we performed acid-fast bacilli staining and found that *L. rhamnosus* PMC203, isolated from vaginal fluid, decreased the abundance of red coloration in the macrophage cells indicating the survival of *M. tb* H37Rv at the concentrations of 4.8 × 10^6^ CFU/ml and 1.2 × 10^6^ CFU/ml, while the other strains did not (Fig. 1C).

We also did CFU assay, and the result showed *L. rhamnosus* PMC203 significantly inhibits the growth of H37Rv strain compared to the control group as well as other strains (Fig. 1D).

### 16S rRNA gene sequencing-based identification of isolated probiotics

The probiotic strains isolated from vaginal microbiota was identified taxonomically by 16S rRNA gene sequencing technology (Supplementary Table S2). The analysis result shows the probiotic strain was 99% similar to 16S rRNA sequences of *L. rhamnosus* strains NBRC 3425 and JCM 1136. Additionally, the sequence was similar to other strains of the *Lactobacillus* genus from 99 to 94%. Based on the analysis, it could be predicted that the isolated strain could be a species in the genus *Lactobacillus*. The other probiotic strain isolated from fermented foods were also previously identified by the 16S rRNA gene sequencing technology.

### Whole-genome analysis result of the strain

**Figure 2:**
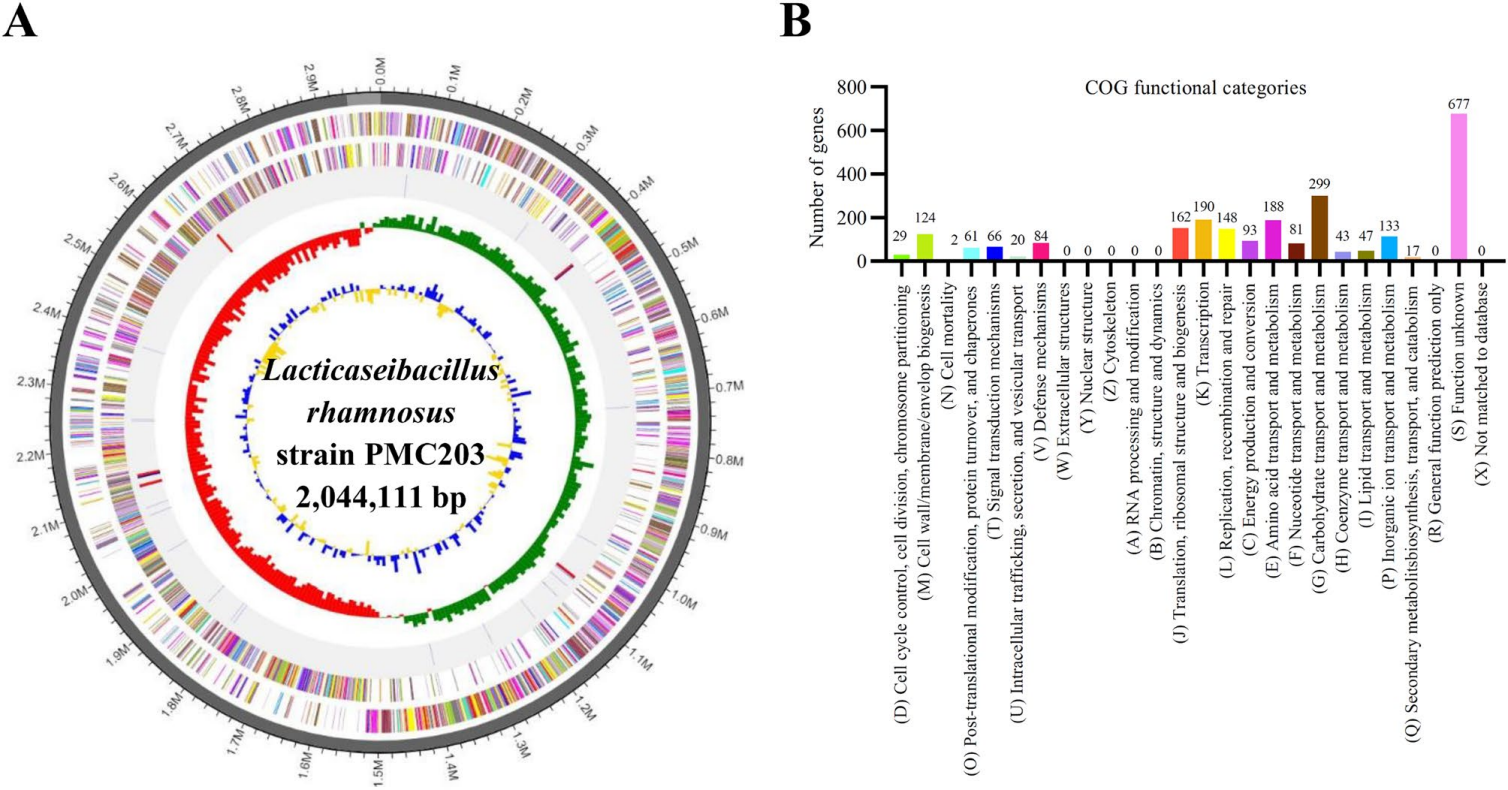
High-throughput genome sequencing results of *L. rhamnosus* strain PMC203. (**A**) Circularmap of *L. rhamnosus* PMC203 strain genome. Antisense and sense strands (colored according to COG categories) and RNA genes (red, tRNA; blue, rRNA) are shown from the outer periphery to the center. Inner circles show the GC skew, with yellow and blue indicating positive and negative values, respectively, and the GC content is indicated in red and green. (**B**) Relative abundance of cluster of orthologous groups (COG) functional categories of genes.

The primary genomic characteristics of PMC203 strain are illustrated in Fig. 2. PMC203 had only one circular chromosome with an average GC content of 46.7 percent and a length of 2,994,218 bp (Fig. 2A). We detected 2785 coding sequences (CDSs) in the genome and the expected CDSs were grouped by the functional categorization system Clusters of Orthologous Groups (COG) (Fig. 2B). From these CDSs, 2434 proteins were assigned to families of COG. Biological functions were identified for 1757 proteins, while 677 CDSs were homologous to preserved protein counterparts whose role was unknown in other organisms. The other 351 hypothetical proteins were not found to correspond to any known proteins in the data base. Furthermore, 60 t-RNA and 15 r-RNA genes were anticipated. The accession number of PMC203 in the NCBI is PRJNA776987.

### OrthoANI genomic similarity

**Figure 3:**
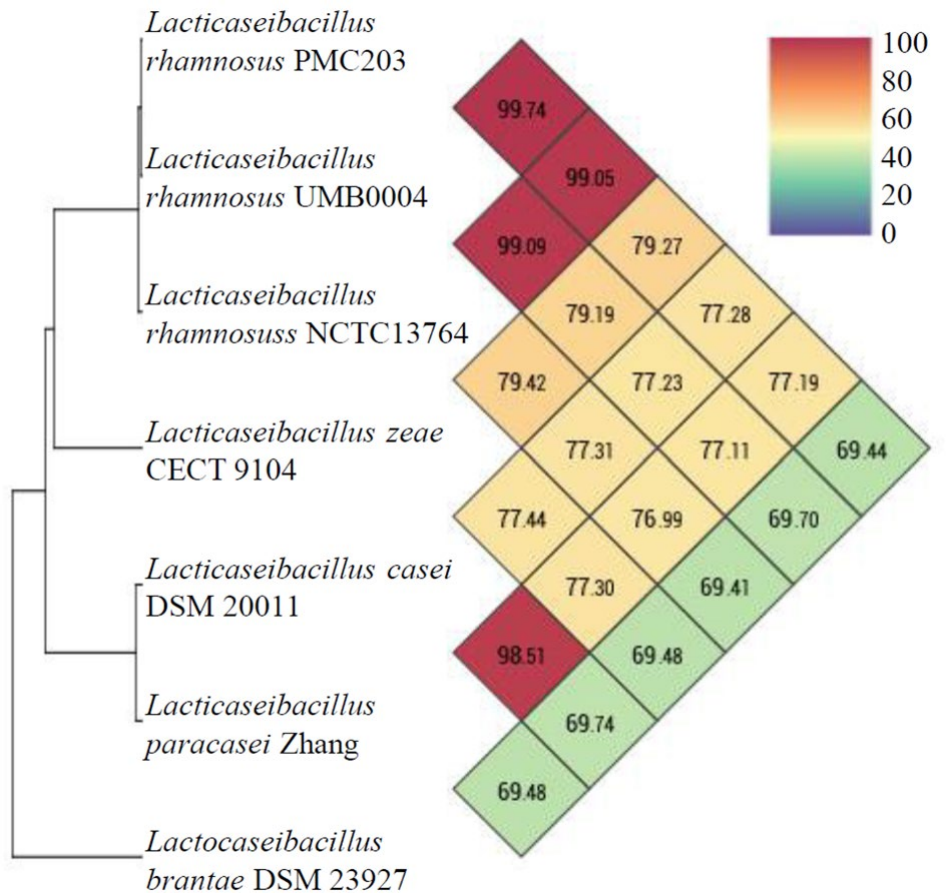
OrthoANI results calculated from available genomes of *Lacticaseibacillus* species. The OrthoANI value of *L. rhamnosus* PMC203 and *L. rhamnosus* UMB0004 was 99.74, higher than 96.0%, the standard for determining the same species.

The OrthoANI approach was used to do similarity analysis for strains with substantial similarities in 16S rRNA analyses utilizing the whole genome sequencing data of PMC203 (Fig. 3). After comparing with PMC203, isolated from this study to all publicly available *Lacticaseibacillus rhamnosus* genomes, we confirmed the similarities of two other strains of *Lacticaseibacillus* UMB0004 and NCTC13764, to be at 99.74 and 99.05 percent, respectively, significantly above the cutoff value of 95% for species delineation^30^. We also compared our strain to other *Lacticaseibacillus* species, including *zeae, casei, paracasei, and brantae*, which had similarities at 79.27 percent, 77.28 percent, 77.19 percent, and 69.44 percent, respectively which were all below 80%. These results strongly suggest that the newly discovered strain PMC203 is *L. rhamnosus.*

### Comparison of genomic characteristics with different strains of *Lacticaseibacillus* species

To investigate whether our isolated strain is newly discovered, we compared the genomic information of PMC203 to other strains of *L. rhamnosus* such as BFE5264^31^, LRB^32^, BPL5^33^, DSM 14870^34^, and JL-1^35^ (Supplementary Table S3). Although they belong to the same species, their source, genome size, G + C content, CDS (coding sequence), r-RNA, and t-RNA numbers differed. These results reveal that PMC203 strain is a newly discovered novel strain of *L. rhamnosus*.

### Intracellular anti-mycobacterial activity of PMC203

**Figure 4:**
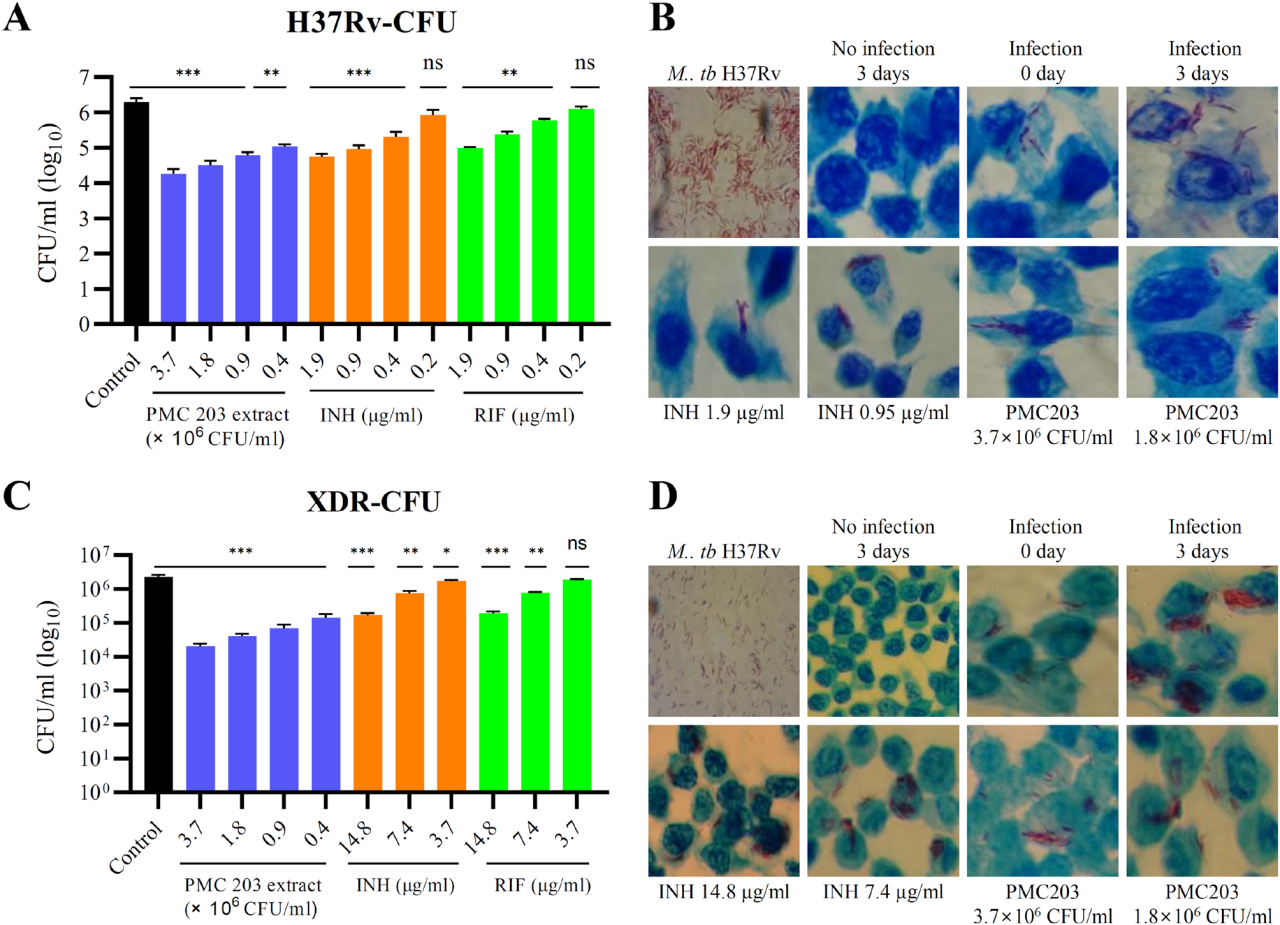
Intracellular killing effect of PMC203 in *M. tb* infected macrophages. Macrophages were infected with (**A**,**B**) *M. tb* H37Rv and (**C**,**D**), XDR *M. tb*, treated with PMC203 cell extract along with control drugs, and incubated 3 days. After incubation, viable bacilli were calculated by (**A**,**C**) CFU enumeration assay and observed by a light microscope after (**B**,**D**) acid-fast bacilli staining. These experiments were carried out in triplicate. Values are expressed as mean values and standard deviations. *Statistical significance with controls was analyzed using Graph Pad Prism 9.1.1, one-way ANOVA (*p < 0.05, **p < 0.01, ***p < 0.001, ****p < 0.0001).

The inhibitory effect of PMC203 on *M. tb* replication in RAW 264.7 cells (Fig. 4) was investigated. Confluent macrophage cell layers grown overnight were infected with H37Rv (A, B) and XDR (C, D), treated with PMC203 extract and reference drugs, isoniazid (INH) and rifampicin (RIF), at a wide range of concentrations, and incubated for 3 days. After incubation, the inhibitory effect of the candidate probiotic strain was analyzed by the CFU method (A, C) and AFB staining method (B, D) through comparison with control sample not treated with anything. PMC203 at 4 × 10^5^ CFU/ml to 3.7 × 10^6^ CFU/ml significantly reduced the titer of *M. tb* H37Rv (Fig. 4A). This effect was similar to the reference drugs at 0.4–1.9 µg/ml. The AFB staining result also showed the anti-tuberculosis effect in which the number of H37Rv was increased in the control sample, whereas reduction of H37Rv was observed in samples treated with PMC203 and reference drugs (Fig. 4B). PMC203 also showed an intracellular inhibition activity on XDR *M. tb* in a macrophage cell line in a dose-dependent manner (Fig. 4C,D). The effect of PMC 203 on XDR *M. tb* was similar to that on *M. tb* H37Rv after 3 days of incubation at 4 × 10^5^ CFU/ml to 3.7 × 10^6^ CFU/ml (Fig. 4C). Reference drugs at 7.4 µg/ml and 14.8 µg/ml showed similar effect to PMC203, but at a 3.7 µg/ml concentration did not show a significant anti-tuberculosis effect. This result indicates that the intracellular activity of PMC203 against XDR is superior to INH and RIF. The anti-mycobacterial effect of PMC203 on XDR *M. tb* was also confirmed through the staining method (Fig. 4D).

### Anti-mycobacterial activities of PMC203 against *M. tb* strains

**Figure 5:**
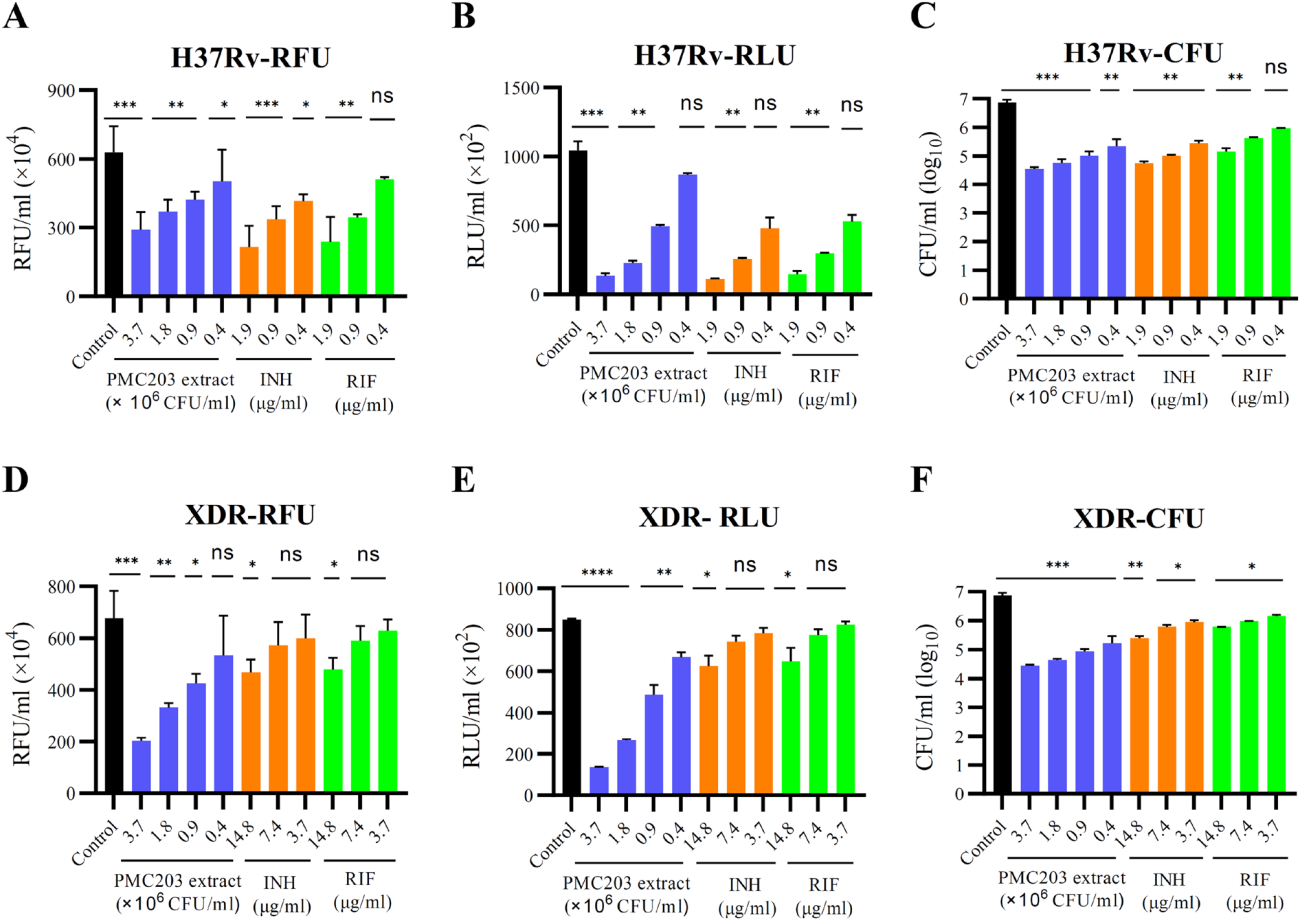
Antimicrobial activities of PMC203 against *M. tb.* The inhibitory effect of PMC203 extract against (**A**–**C**) H37Rv or (**D**–**F**) XDR strains was evaluated. Mycobacterial susceptibility was checked by (**A**,**D**) resazurin assay as RFU/ml (relative fluorescence unit per ml), by (**B**,**E**) microbial cell viability assay as RLU/ml (relative luminescence unit per ml), and by (**C**,**F**) CFU quantification assay as CFU/ml. These experiments were performed three times in triplicate. Values are expressed as mean values and standard deviations. *Statistical significance with controls were determined using Graph Pad Prism 9.1.1, one-way ANOVA (*p < 0.05, **p < 0.01, ***p < 0.001, ****p < 0.0001).

Anti-mycobacterial activities of PMC203 extract against *M. tb* strains were assessed (Fig. 5). The assay result showed the significant growth inhibition of *M. tb* H37Rv in broth medium at a PMC203 dose level of 4 × 10^5^ CFU/ml to 3.7 × 10^6^ CFU/ml (Fig. 5A–C). Reference drugs also showed a significant effect against H37Rv at lower concentrations ranging from 0.4 to 1.9 µg/ml. The anti-mycobacterial effect of PMC203 extract against XDR *M. tb* was also evaluated, which shows a similar effect like that on *M. tb* H37Rv, but the reference drugs required much higher concentration than that on *M. tb* H37Rv (Fig. 5D–F). This result further confirms the anti-mycobacterial superiority of PMC203 extract against XDR *M. tb* to reference drugs INH and RIF. These findings indicate that PMC203 extract has anti-tuberculosis activities against drug-sensitive and drug-resistant *M. tb* strains*.*

### Activity of PMC203 against H37Rv in co-culture assay

**Figure 6:**
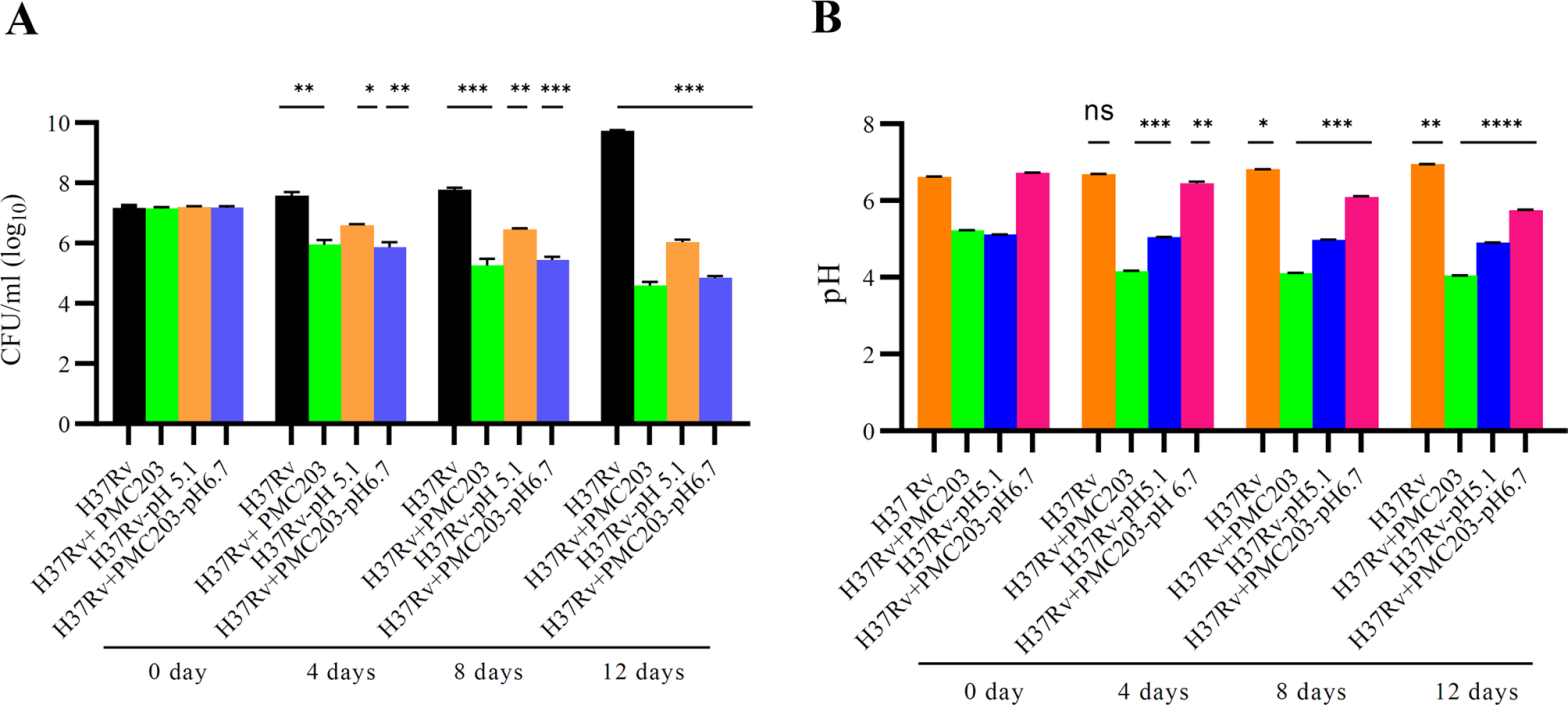
The activity of PMC 203 against *M. tb* H37Rv in Co-culture condition. Mycobacterial cells in different conditions were grown in vitro in the presence of PMC203 for 12 days. Viable bacilli were enumerated based on (**A**) CFU/ml and (**B**) pH changes were also checked during the treatment period. *Statistical significances with controls were determined in Graph Pad Prism 9.1.1 using one-way ANOVA (*p < 0.05, **p < 0.01, ***p < 0.001).

In broth co-culture conditions, the capacity of PMC203 to suppress the growth of *M. tb* H37Rv was tested (Fig. 6). On days 0, 4, 8, and 12, the CFU of *M. tb* (Fig. 6A) and the broth pH (Fig. 6B) were assessed when growing *M. tb* alone or combined with PMC203. *M. tb* single culture and co-culture with PMC203 had initial pH values of 6.6 and 5.2, respectively. After 12 days of incubation, the former climbed to 5.4 × 10^9^ CFU/ml (p < 0.001) and pH 6.9 (p < 0.01), while the latter declined to 3.9 × 10^4^ CFU/ml (p < 0.001) and pH 4.04 (p < 0.0001). Furthermore, the *M. tb* culture adjusted to an initial pH of 5.1 became 1.07 × 10^6^ CFU/ml (p < 0.001) and pH 4.9 (p < 0.0001), while the co-culture adjusted to an initial pH of 6.7 became 6.9 × 10^4^ CFU/ml (p < 0.001) and pH 5.7 (p < 0.0001), which was a decrease than the single culture.

### Cytotoxicity of PMC203

**Figure 7:**
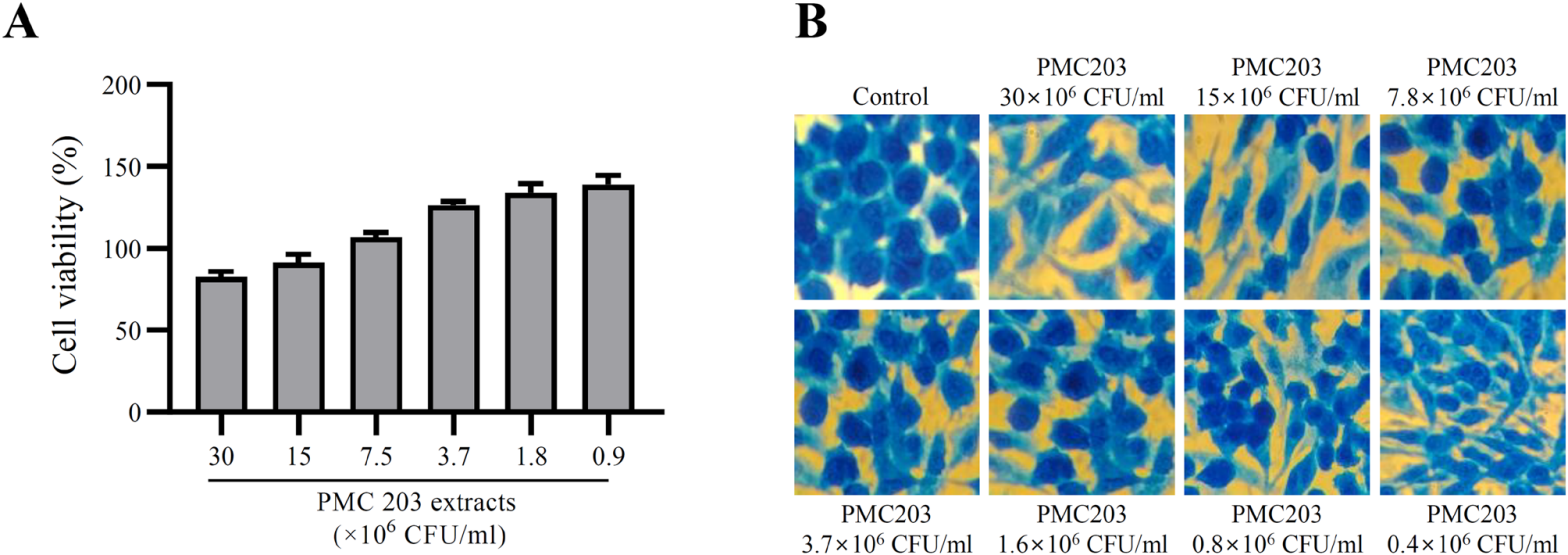
Cell cytotoxicity evaluation of PMC203 on macrophage cell lines. Cytotoxicity of PMC203 cell extract was evaluated at a wide range of concentrations against macrophage Raw 264.7 cell lines by (**A**) Ez-cytox, viability of cells were also evaluated by (**B**) methylene blue staining. Experiments were performed three times in triplicate. Values are expressed as mean values and standard deviations.

The cytotoxicity of PMC203 is shown in Fig. 7. The result showed that the PMC203 extract had no significant cytotoxicity up to a concentration of 30 × 10^6^ CFU/ml (Fig. 7A) using Ez-cytox reagent. We also determined the cell viability using a trypan blue staining test in which treated cells were observed under an optical microscope (Fig. 7B). No apparent decrease of viability or morphological changes were observed compared to the control sample at the similar concentration of PMC203 extract used in the Ez-cytox cell viability assay test.

### Repeated oral toxicity assay of PMC203 in guinea pig

The oral-dose toxicity of PMC203 was assessed in guinea pigs (Supplementary Fig. S1). The probiotic strain was regularly orally administered for 2 weeks, and acute toxicity was examined during the entire study period. There was no significant difference in body weight between guinea pigs treated with PMC203 and guinea pigs treated with 0.85% NaCl solution. No remarkable clinical observations or death were seen in the animals after treating them with the probiotic strain. The symptoms observed are listed in the Supplementary Table S5. Furthermore, treatment with PMC203 did not affect the food and water consumption pattern during the trial. Our findings imply that guinea pigs are not harmed by acute exposure to PMC203.

### PMC203 stimulates autophagy in RAW 264.7 cells

**Figure 8:**
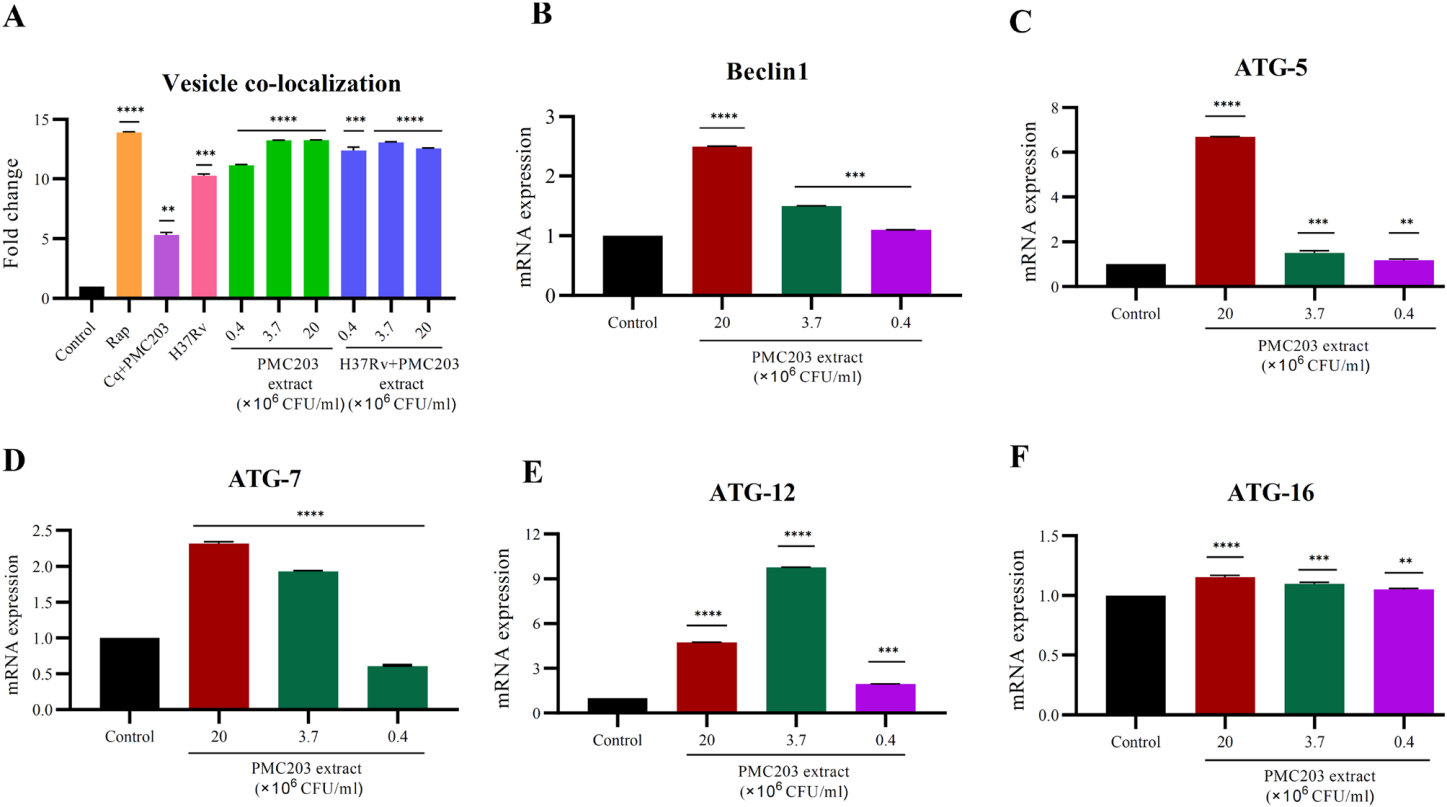
Induction of autophagy by PMC203. (**A**) Raw 264.7 cell lines were treated with the cell extract of PMC203 at three different concentrations and then vesicle co-localization with LC3 signal was measured using autophagy detection kit by fluorescence microplate reader. (**B**–**F**) The mRNA expressions of Beclin1 pathway, and autophagy gene complex was detected by quantitative Real-Time PCR. These experiments were performed three times in triplicate. Values are expressed as mean values and standard deviations. *Statistical significance with controls were determined using Graph Pad Prism 9.1.1, one-way ANOVA (*p < 0.05, **p < 0.01, ***p < 0.001, ****p < 0.0001). *Rap* rapamycin, *Cq* chloroquine.

To determine whether PMC203 induces autophagy, RAW 264.7 cells were seeded, treated with predetermined concentrations of the probiotic strain, and incubated (Fig. 8). For cells treated with PMC203 (3.7 × 10^6^ CFU/ml and 20 × 10^6^ CFU/ml) or 1 µM autophagy activator rapamycin (Rap)^36^, the green detection reagent signal was increased significantly (p < 0.0001) compared to untreated cells. The increasing trend of green detection signal indicates the intensity of autophagy induction^37^. In the cells infected with H37Rv, the signal was increased 10.2 times. When PMC203 was treated in the infected cells, the signal was further increased to 12.4–13.1 times (Fig. 8A). To further confirm the autophagy induction by PMC203, macrophage cells were treated with autophagy inhibitor chloroquine (Cq)^38^ for 5 h before probiotic treatment, and we found that the green detection signal was not increased like the cells treated with PMC203 alone (2 × 10^7^ CFU/ml). Moreover, the mRNA expression of beclin1, *Atg-5*, *Atg-7*, *Atg-12*, and *Atg-16* has been markedly increased (p < 0.0001) at 2 × 10^7^ CFU/ml or 3.7 × 10^6^ CFU/ml of PMC203 (Fig. 8B–F).

## Discussion

*M. tb*, which infects one-third of the world's population, considering one of the most effective bacteria among those that cause infectious diseases^39^. Besides, its innate capacity to survive the host’s defensive mechanisms, *M. tb* can withstand the majority of antimicrobial agents currently available due to the sequential accumulation of resistance mutations, emerging as MDR, XDR, and most recently TDR^40,41^. To combat this alarming situation of drug resistant TB, different approaches are being proposed, for example, repurposed drugs as combinational therapy, pathogen-centric strategies for developing new compounds with a different mechanism of action, and host-direct therapeutics that modulate the host immune response^42^. In these circumstances, probiotics can be an alternative for TB treatment as probiotics have recently been highlighted for their potential roles in controlling tuberculosis through the stimulation of host immunoglobulins and antibacterial compounds^43,44^. As part of alternative TB treatment, we investigated the anti-tuberculosis effect of probiotic strain *L. rhamnosus* PMC203, isolated from vaginal microbiota. We also explored the possible underlying mechanism of intracellular killing of our candidate probiotic strain.

Probiotic strain PMC203, isolated from vaginal microbiota, was determined to be *L. rhamnosus* according to the similarity cutoff criteria of 98.65% based on 16S rRNA gene sequence and 95% based on whole-genome sequence^30,45^. Furthermore, it was also identified as a newly discovered strain since its chromosomal characteristics were different from other strains of *L. rhamnosus*. *L. rhamnosus* is now known as *Lacticaseibacillus rhamnosus* in the scientific community^46^.

The primary site of TB infection is the lungs. A study reported that pulmonary disease is present in more than 80% of TB cases^47^. Once the bacterium, cluster, or clump is delivered in a water droplet into an alveolus and ingested by an alveolar macrophage, the infection begins^48^. As RAW 264.7 macrophage cell lines have been used extensively as an in vitro model in tuberculosis research^49,50^, we also used the same cell lines to investigate the intracellular killing effect of our candidate probiotic strain. PMC203 showed an effect against drug-sensitive and drug resistant tuberculosis at a concentration that did not show cytotoxicity in macrophage cell lines. In our study, instead of live bacteria, we used probiotic lysate that can be used as adjunctive therapy with TB drugs without worrying about the viability in the antibiotic environment. Nowadays, many researchers are reporting the use of probiotics as new adjuvants in the treatment of various diseases^51–55^ and our candidate probiotic lysate can also be used in this context as adjunctive therapy with TB medications.

TB can also affect any part of the body. Extrapulmonary TB occurs in about 15–20% of TB infection cases but can be seen in more than 50% of cases in immunocompromised patients with HIV^56,57^. The gastrointestinal TB is the most commonly affected among the extrapulmonary TB infection sites, accounting for 3–5% of all extrapulmonary TB cases^58^. PMC203 showed significant anti-tuberculosis effect in the anti-mycobacterial susceptibility assay and co-culture experiment, so it is considered to apply to the extrapulmonary tuberculosis model. However, this experiment has a limitation in that it is difficult to apply to the pulmonary tuberculosis model.

*L. rhamnosus* is considered to be a GRAS (Generally Regarded as Safe) bacteria^59^ that has numerous therapeutic properties^60^. *L. rhamnosus* has also been granted Qualified Presumption of Safety (QPS) status by the European Food Safety Authority (EFSA), indicating the use of this microorganism in the production of food or feed raises no safety concerns^61^. It also has a long documented tradition of safe use in cheesemaking^62^. Though probiotics are generally regarded as safe, there are reports of toxicity such as sepsis, particularly in immunocompromised patients^63^. Taking these safety issues as a reminder that this agent may also cause disease, we conducted a 2-week repeated oral administration toxicity test using guinea pig, and we did not observe any noticeable clinical symptoms during the study period. The candidate probiotic strain also did not show cytotoxicity on macrophage cell lines.

*Autophagy* is an elaborate cellular process where cytoplasmic targets are captured in double-membrane autophagosomes and then transported to lysosomes for degradation^64^. It is an essential part of the immune defense against pathogenic bacteria like *Mycobacterium tuberculosis*^65^. From this background, we investigated whether PMC203 is involved in autophagy induction. Based on our autophagy detection assay, vesicle co-localization is increased by PMC203 treatment. This result suggests that PMC203 induces microtubule-associated protein 1 light chain 3 (LC3) protein, a marker widely used to monitor autophagy^66^. To examine the underlying mechanisms of PMC203 induced autophagy, we observed the effect of PMC203 on the autophagic signaling pathway, beclin1; beclin1 is a core protein in autophagosome nucleation that enables the recruitment of several autophagy proteins involved in the nucleation of autophagosomes^67^. We also determined the mRNA expression of autophagy-related gene families called ATG (AuTophaGy-related gene), the core of the molecular machinery of the autophagy complex^68^, which is essential to LC3 ligation to the autophagosome membrane^69^. Our study results suggest that beclin 1 and the ATG gene complex activate PMC203-induced autophagy to reduce the *M. tb* burden in macrophage cell lines that is similar to the previously published study.

Despite the experimental results on PMC203 at the in vitro level, extensive additional studies such as long-term oral toxicity test, in vivo efficacy evaluation, clinical trial and synergistic effect evaluation with tuberculosis drugs are needed.

In conclusion, we isolated a new strain *L. rhamnosus* PMC203 from the vaginal microbiota that showed effects on both drug-sensitive and drug-resistant *M. tb* strains in the macrophage RAW 264.7 cell line. These findings suggest its potentiality to be used as an anti-tuberculosis drug candidate for treating both drug sensitive and drug resistant tuberculosis. However, other extensive studies are still needed to conduct the next phase of experiments necessary for the development of new anti-tuberculosis drugs.

## Supplementary Information

Supplementary Information.

## Data Availability

The whole genome sequencing data of *Lacticaseibacillus rhamnosus* PMC203 is now available in the following web link: https://www.ncbi.nlm.nih.gov/nuccore/CP086326.1.
