# Supplementary material for: In vitro anti-tuberculosis effect of probiotic Lacticaseibacillus rhamnosus PMC203 isolated from vaginal microbiota
Source: Sci Rep. 2022 May 18;12:8290. doi: 10.1038/s41598-022-12413-z (PMC9116076; doi:10.1038/s41598-022-12413-z)
Supplement: Supplementary file 1 — Supplementary Information. [file 41598_2022_12413_MOESM1_ESM.pdf]

In vitro anti-tuberculosis effect of probiotic *Lacticaseibacillus rhamnosus* PMC203 isolated from vaginal microbiota.

Md Abdur Rahim[1,2], Hoonhee Seo[1], Sukyung kim[1], Hanieh Tajdojian[1,2], Indrajeet Barman[1,2], Youngkyoung Lee[1,2], Saebim Lee[1], and Ho-Yeon Song[1,2]

[1]Probiotics Microbiome Convergence Center, Soonchunhyang University, Asan, Chungnam, 31538, Korea

[2]Department of Microbiology and Immunology, School of Medicine, Soonchunhyang University, Cheonan, Chungnam, 31151, Korea

Md Abdur Rahim and Hoonhee Seo contributed equally to this study.

*Correspondence to: Ho-Yeon Song

E-mail: songmic@sch.ac.kr

Supplementary Information

Supplementary Table S1. List of test candidate probiotics used in this study

Sl. No.	Organism's name	Sources
1	*Lactobacillus sakei (L. sakei)*	Fresh kimchi
2	*Staphylococcus erucrum (S. erucrum)*	Curved cheese
3	*Lactobacillus curvatus (L. curvatus)*	Radish kimchi
4	*Lactobacillus pentosus (L. pentosus)*	Sesame leaf kimchi
5	*Weissella cibaria (W. cibaria)*	Young radish kimchi
6	*Lactobacillus paracasei (L. paracasei)*	Curved kimchi
7	*Weissella cibaria (W. cibaria)*	Sesame leaf kimchi
8	*Leuconostoc citreum (L. citreum)*	Radish kimchi
9	*Lactobacillus sakei (L. sakei)*	Young radish kimchi
10	*Leuconostoc lactis (L. lactis)*	White kimchi
11	*Lactobacillus graminis (L. graminis)*	Radish kimchi
12	*Bacillus amyloliquefaciens (B. amyloliquefaciens)*	Vinegar
13	*Leuconostoc mesenteroides (L. mesenteroides)*	White kimchi
14	*Lactobacillus rhamnosus (L. rhamnosus)*	Vaginal fluid (SCH hospital)
15	*Bacillus velezensis (B. velezensis)*	Old Kimchi
16	*Bacillus subtilis (B. subtilis)*	Old kimchi
17	*Acetobacter ascendens (A. ascendens)*	Vinegar
18	*Lactobacillus delbrueckii (L. delbrueckii)*	Vaginal fluid (SCH hospital)
19	*Lactobacillus curvatus (L. curvatus)*	Fresh kimchi
20	*Lysinihacillus macrolides (L. macrolides)*	Old kimchi

Supplementary Table S2. Identification of isolated bacterial strain, PMC203 based on 16S rRNA gene analysis

NCBI Ref	Organisms	Length	Score	Identities	Gaps
NR_113332.1	*Lactobacillus rhamnosus* strain NBRC 3425	1495	2676 bits (1449)	1454/1456 (99%)	1/1456 (0%)
NR_043408.1	*Lactobacillus rhamnosus* strain JCM 1136	1521	2652 bits (1436)	1446/1452 (99%)	2/1452 (0%)
NR_037122.1	*Lactobacillus zeae* strain RIA 482	1522	2601 bits (1408)	1440/1456 (99%)	1/1456 (0%)
NR_041893.1	*Lactobacillus casei* subsp. Casei ATCC 393	1517	2590 bits (1402)	1444/1464 (99%)	4/1464 (0%)
NR_025880.1	*Lactobacillus paracasei* strain R094	1522	2582 bits (1398)	1437/1456 (99%)	1/1456 (0%)
NR_113337.1	*Lactobacillus paracasei* strain NBRC 15889	1495	2584 bits (1399)	1437/1456 (99%)	1/1456 (0%)
NR_113333.1	*Lactobacillus casei* strain NBRC 15883	1495	2580 bits (1397)	1437/1457 (99%)	3/1457 (0%)
NR_113823.1	*Lactobacillus paracasei* subsp. strain NBRC 15906	1497	2571 bits (1392)	1436/1457 (99%)	3/1457 (0%)
NR_117987.1	*Lactobacillus paracasei* strain ATCC 25302	1441	2536 bits (1373)	1415/1435 (99%)	3/1435 (0%)
NR_115322.1	*Lactobacillus casei* strain BCRC10697	1528	2558 bits (1385)	1441/1467 (98%)	7/1467 (0%)
NR_115534.1	*Lactobacillus casei* subsp. casei ATCC 393	1522	2575 bits (1394)	1437/1458 (99%)	5/1458 (0%)
NR_115322.1	*Lactobacillus casei* strain BCRC10697	1528	2558 bits (1385)	1441/1467 (98%)	7/1467 (0%)
NR_125575.1	*Lactobacillus brantae* DSM 23927 strain SL1108	1545	2333 bits (1263)	1400/1465 (96%)	13/1465 (1%)

Supplementary Table S3. Comparison of chromosomal properties of *Lactocaseibacillus rhamnosus* PMC203 strains.

Strain	PMC203	BFE5264	LRB	BPL5	DSM 14870	JL-1
Sources	Vaginal fluid	Maasai	Healthy baby tooth	Vagina	Vaginal epithelial cells	Infant feces
Genome size (bp)	2,994,218	3,068,152	2,934,954	3,024,027	3,013,149	3,007,502
G+C content (%)	46.7	46.8	46.8	46.7	46.7	46.7
Predicted CDS	2,785	3,077	2,428	2,751	1677	2,745
Number of rRNA genes	15	15	15	15	5	15
Number of tRNA genes	60	60	59	59	55	59

Supplementary Table S4. Primer pair sequences used in this study

Primer name	Sequences
Beclin1 (F)	GCCTCTGAAACTGGACACGA
Beclin1 (R)	CTTCCTCCTGGCTCTCTCCT
ATG 5 (F)	AGAAGATGTTAGTGAGATATGG
ATG 5 (R)	ATGGACAGTGTAGAAGGT
ATG 7 (F)	AGCCCACAGATGGAGTAGCAGTTT
ATG 7 (R)	TCCCATGCCTCCTTTCTGGTTCTT
ATG 12 (F)	CCAAGGACTCATTGACTTC
ATG 12 (R)	GCAAAGGACTGATTCACATA
ATG 16 (F)	TGTCTTCAGCCCTGATGGCAGTTA
ATG 16 (R)	AGCACAGCTTTGCATCCTTTGTCC
GAPDH (F)	TCCCATCACCATCTTCCA

Supplementary Table S5. Summary of clinical signs during the acute oral toxicity test

Group	No. of animals	Clinical sign	Days after dosing													
			1	2	3	4	5	6	7	8	9	10	11	12	13	14
Control	5	No stool	0	0	0	0	0	0	0	0	0	0	0	0	0	0
		Mucous stool	0	0	0	0	0	0	0	0	0	0	0	0	0	0
		Soft stool	0	0	0	0	0	0	0	0	0	0	0	0	0	0
		Refusal to feed	0	0	0	0	0	0	0	0	0	0	0	0	0	0
		Decrease in food intake	0	0	0	0	0	0	0	0	0	0	0	0	0	0
		Emaciation	0	0	0	0	0	0	0	0	0	0	0	0	0	0
		Loss of fur	0	0	0	0	0	0	0	0	0	0	0	0	0	0
		Salivation (after dosing)	0	0	0	0	0	0	0	0	0	0	0	0	0	0
		Lying on side	0	0	0	0	0	0	0	0	0	0	0	0	0	0
		Death	0	0	0	0	0	0	0	0	0	0	0	0	0	0
		NOA	5	5	5	5	5	5	5	5	5	5	5	5	5	5
PMC203 (2×10^6 CFU/ml)	5	No stool	0	0	0	0	0	0	0	0	0	0	0	0	0	0
		Mucous stool	0	0	0	0	0	0	0	0	0	0	0	0	0	0
		Soft stool	0	0	0	0	0	0	0	0	0	0	0	0	0	0
		Refusal to feed	0	0	0	0	0	0	0	0	0	0	0	0	0	0
		Decrease in food intake	0	0	0	0	0	0	0	0	0	0	0	0	0	0
		Emaciation	0	0	0	0	0	0	0	0	0	0	0	0	0	0
		Loss of fur	0	0	0	0	0	0	0	0	0	0	0	0	0	0
		Salivation (after dosing)	0	0	0	0	0	0	0	0	0	0	0	0	0	0
		Lying on side	0	0	0	0	0	0	0	0	0	0	0	0	0	0
		Death	0	0	0	0	0	0	0	0	0	0	0	0	0	0
		NOA	5	5	5	5	5	5	5	5	5	5	5	5	5	5

NOA, No observable abnormality

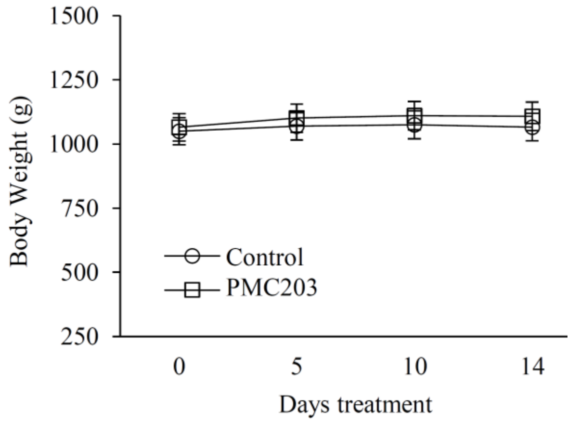

Supplementary Figure S1. Changes in body weight of guinea pig during acute oral toxicity study for 2 weeks. Control group was provided only saline water while treatment group was provided saline water containing PMC203 at the dose level of 2×10^{8} CFU/ml. All guinea pig were weighed on days 0, 5, 10, and 14.
